# Size-Sorted Superheated Nanodroplets for Dosimetry and Range Verification of Carbon-Ion Radiotherapy

**DOI:** 10.3390/nano14201643

**Published:** 2024-10-13

**Authors:** Yosra Toumia, Marco Pullia, Fabio Domenici, Alessio Mereghetti, Simone Savazzi, Michele Ferrarini, Angelica Facoetti, Gaio Paradossi

**Affiliations:** 1 National Institute for Nuclear Physics (INFN), sez. Roma Tor Vergata, 00133 Rome, Italy; fabio.domenici@uniroma2.it (F.D.); gaio.paradossi@uniroma2.it (G.P.); 2 Department of Chemical Science and Technologies, University of Rome Tor Vergata, 00133 Rome, Italy; 3 Fondazione CNAO, National Center for Oncological Hadrontherapy, 27100 Pavia, Italy; marco.pullia@cnao.it (M.P.); alessio.mereghetti@cnao.it (A.M.); simone.savazzi@cnao.it (S.S.); michele.ferrarini@cnao.it (M.F.); angelica.facoetti@cnao.it (A.F.)

**Keywords:** nanodroplets, ultrasound imaging, carbon-ions radiotherapy, dosimetry, range verification

## Abstract

Nanodroplets have demonstrated potential for the range detection of hadron radiotherapies. Our formulation uses superheated perfluorobutane (C4F10) stabilized by a poly(vinyl-alcohol) shell. High-LET (linear energy transfer) particles vaporize the nanodroplets into echogenic microbubbles. Tailored ultrasound imaging translates the generated echo-contrast into a dose distribution map, enabling beam range retrieval. This work evaluates the response of size-sorted nanodroplets to carbon-ion radiation. We studied how thesize of nanodroplets affects their sensitivity at various beam-doses and energies, as a function of concentration and shell cross-linking. First, we show the physicochemical characterization of size-isolated nanodroplets by differential centrifugation. Then, we report on the irradiations of the nanodroplet samples in tissue-mimicking phantoms. We compared the response of large (≈900 nm) and small (≈400 nm) nanodroplets to different carbon-ions energies and evaluated their dose linearity and concentration detection thresholds by ultrasound imaging. Additionally, we verified the beam range detection accuracy for the nanodroplets samples. All nanodroplets exhibited sensitivity to carbon-ions with high range verification precision. However, smaller nanodroplets required a higher concentration sensitivity threshold. The vaporization yield depends on the carbon-ions energy and dose, which are both related to particle count/spot. These findings confirm the potential of nanodroplets for range detection, with performance depending on nanodroplets’ properties and beam parameters.

## 1. Introduction

Ionizing radiation therapy with heavy particles such as protons and carbon ions (hadron therapy) has gained significant interest in recent years as an alternative to conventional photon radiotherapy for treating resistant tumors. The charged particle radiation is characterized by the Bragg peak (BP), a localized sharp distal fall-off where the particles deliver the maximal amount of their energy before stopping. This characteristic peak allows for a high dose of radiation to be precisely targeted at the tumor while sparing the surrounding healthy tissue [1]. Although still growing and less available than protons, (with only 15 clinical facilities operating to date all over the world [2]), carbon ion (C-ion) radiotherapy is more effective. C-ions are heavier than protons and can release a higher dose to tumors with minimized exposure of the healthy tissue. In addition, C-ions feature a higher linear energy transfer (LET), leading to increased radiobiological effectiveness (RBE) in damaging cancer cells, thereby improving treatment outcomes and reducing side effects [3,4,5].

However, addressing uncertainties remains crucial for delivering the desired treatment plan. In this regard, in situ dosimetry and range verification in patients are key challenges. The uncertainties in dose distribution during treatment delivery arise from clinical considerations related to setup errors or anatomical variations due to patient motion (e.g., breathing) or changes in the tumor size and shape throughout the treatment. These errors can jeopardize the precision of target localization delivery, potentially leading to under-dosage within the tumor and/or over-dosage in the neighboring healthy tissue. 

Range verification methods aim to compensate for errors and ensure accurate treatment by reducing safety margins. These methods include medical imaging techniques such as prompt gamma imaging, positron emission tomography (PET) and computed tomography (CT) [6,7,8,9,10]. Each approach presents advantages and limitations in terms of applicability in real-time, setup complexity, and cost. Often, combining multiple range verification techniques is recommended to achieve the highest level of accuracy. For example, Sun et al. showed the feasibility of PET/CT for clinical dose verification after C-ion irradiation [11]. While offline methods can be effective, in-beam range verification is ideal and highly desirable because it allows for immediate corrections if there are discrepancies between the planned and delivered dose distribution. Clinical trials are currently being conducted using an online qualitative monitoring system at the National Center of Oncological Hadrontherapy (CNAO, Pavia, Italy) [12,13,14]. This system combines a detector that collects signals from the emitted charged particles with a PET scanner, enabling range verification via short-living positron emitters generated by the interaction of C-ions with the tissue. However, further efforts to improve data processing are still needed. 

Recently, we developed a non-invasive injectable tracer for intravenous administration that has the potential to simultaneously allow dosimetry and range verification of hadron therapy (protons and C-ions) with very low uncertainty by using medical ultrasound (US) imaging. Specifically, this method involves superheated nanodroplets (NDs) that vaporize into echogenic microbubbles (MBs) upon interaction with the ionizing radiation. Following this liquid-to-gas phase transition, real-time US imaging translates the generated echo-contrast from MBs into a dose distribution and LET combination map enabling the retrieval of the beam range [15,16,17]. NDs, also known as phase-shift US contrast agents, are generally sub-micron particles consisting of a liquid perfluorocarbon (PFC) core encapsulated in a lipid or polymer shell that were originally developed to overcome some restrictions in conventional MBs, such as bloodstream circulation lifetime and enhanced permeability and retention effect (EPR) [18]. Besides US imaging, NDs could be further engineered as targeted drug carriers or multi-modal imaging platforms [19,20,21,22,23]. Typically, NDs generate ultrasound contrast only when they undergo transition from a liquid to a gas state upon external stimulation, such as heating, acoustic/optical droplet vaporization processes (ADV/ODV), and ionizing particles. The boiling temperature of the PFC core determines NDs stability/reactivity against the external stimuli. Nonetheless, the stability of NDs is also affected by Laplace pressure which is a function of the interfacial tension within the droplet and its radius (see Equation (1)), and therefore depends on NDs’ size and the shell properties [18].

(1) $$\Delta P=P_{\mathrm{in}}\;-\;P_{\mathrm{out}}=\frac{2\sigma }{r}$$

where ΔP, P_in_, and P_out_ are the Laplace pressure, the pressure inside, and outside the core, respectively, σ is the surface tension at the nanodroplet interface, and *r* is the radius of the nanodroplet.

The concept of using NDs for radiotherapy dosimetry is inspired from superheated drop detectors. These detectors are based on emulsions of a halocarbon stabilized at a temperature above its boiling point in aqueous gel or soft polymer matrix [24]. The LET threshold of the charged particles, i.e., the amount of energy required to be deposited by the particles per unit of length to induce nucleation of the metastable drops, strongly depends on the reduced superheat degree of these latter, defined by Equation (2):

(2) $$s=\frac{T-T_{b}}{T_{c}-T_{b}}$$

where *T_b_* is the boiling temperature of the considered liquid, *T_c_* is the critical temperature, and *T* is the experimental temperature during radiation exposure.

Bearing in mind these considerations, designing effective injectable NDs for radiotherapy should prioritize stability at 37 °C in the absence of radiation, sensitivity once exposed to the ionizing particles of interest, and biocompatibility. Furthermore, the small sizes of NDs (<500 nm), combined with their chemical versatility, are an asset for theranostic applications. The NDs described herein are formulated starting from approved compounds: a superheated perfluorobutane (PFB, b.p. −2 °C) core stabilized with a cross-linked poly(vinyl-alcohol) (PVA) shell. PFB has been used as the gas core for Sonazoid^TM^ MBs (GE Hekathcare, Chicago, IL, USA), approved in Japan, Norway, and Denmark for diagnosing focal liver lesions via contrast-enhanced US (CEUS) imaging, while PVA is a biocompatible synthetic polymer employed in several pharmaceutical emulsions and approved by the FDA and EMA [25,26]. At 37 °C, the calculated reduced superheat degree of PFB according to Equation (1) is ≈0.34. Theoretical assumptions predict a track-averaged LET threshold ≈145 keV/μm to sensitize PFB to charged particles at physiological temperature [16]. C-ion radiotherapy demonstrates an optimal efficacy against resistant tumors when the LET value is about 150–200 keV/μm [27,28], which makes the PFB-based ND system a suitable candidate for C-ion dose distribution detection. It is noteworthy that the sensitivity of NDs to C-ions may be influenced by the size and shell properties (thickness, elastic modulus, viscosity), which confer higher stability to the system.

We have demonstrated the feasibility of hadron therapy dosimetry and range verification through both offline and online US imaging using polydisperse PVA-shelled PFB (PVA/PFB) NDs [15,17]. In proton beams, these PVA/PFB NDs showed a response to high-LET secondary recoils at physiological conditions. However, in C-ion beams, the NDs showed evident responsiveness at the BP with sub-millimeter accuracy. In addition, the use of a PVA shell in the ND formulation provided excellent thermal stability, likely due to the enhanced thickness and covalent cross-links networking the polymer chains compared to the more commonly used thin lipid shells. PVA also offers a functional surface allowing tuning the properties of NDs, making them a versatile platform for multimodal-radiotherapy dosimetry. For instance, drugs like boronophenylalanine (BPA) can be anchored to the surface of NDs for use in boron neutron capture therapy (BNCT) [29]. 

In this manuscript, we expand on previous findings to better understand the mechanism behind C-ion-radiation-induced vaporization of NDs, considering the possible influence of NDs’ size and shell properties on their sensitivity. We increased the reaction time during PVA/PFB ND synthesis to increase shell cross-links and used differential centrifugation to separate large and small NDs from polydisperse suspensions obtained with two reaction times. We then evaluated the response detection of size-sorted ND samples embedded in tissue-mimicking phantoms to C-ion beams under various conditions (dose, energy, concentration) by means of US imaging, illustrating the potential of NDs for range verification even in complex beam settings. Evidence that small-sized NDs are sensitive to C-ion radiation would be very promising for future in vivo studies, not only in terms of accumulation in tumors through the EPR effect but also regarding safety concerns, as large droplets leading to significant bubble inflation after vaporization could pose a risk if the size exceeds the blood capillary diameter.

Finally, we believe that using US imaging with PVA/PFB NDs is an innovative, low-cost and non-invasive approach that could offer, in perspective, an alternative solution for the direct and real-time monitoring of C-ion radiotherapy, facilitating the routine protocols.

## 2. Materials and Methods

### 2.1. Nanodroplets Synthesis

The nanodroplets, made of liquid PFB in the metastable state encapsulated by a cross-linked PVA shell, were prepared as described elsewhere [30]. First, fully hydrolyzed PVA powder (Mn = 30 ± 5 kg/mol; Merck, Milan, Italy) is dissolved at 80 °C in mQ water at a concentration of 2% (*w*/*v*). Then, once the mixture becomes clear after the complete dissolution of PVA, the heating is stopped and NaIO_4_ (Merck, Milan, Italy) oxidant is added at a ratio of 2% (mol: mol) with respect to the PVA repeating unit. The solution is kept under stirring for 1 h to split the head-to-head sequences, leading to telechelic PVA with aldehyde groups as chain terminals, responsible for the networking reaction with the PVA chain’s hydroxyl moiety. PFB (Apollo Scientific, Manchester, UK) is then condensed at low temperature by fluxing it for only a few seconds in an empty glass vial sealed with a rubber septum and immersed in liquid nitrogen. Subsequently, 5 mL of the telechelic PVA aqueous solution is injected into the vial while still immersed in the liquid nitrogen, followed by an immediate sonication of the mixture for 15 min or 25 min at 100% power using an ice-cold ultrasonic bath cleaner (200 W, 40 kHz, Ceia CP104, Florence, Italy). We opted for two sonication durations in this study to further assess the effect of cross-linking efficiency on the ND response to C-ions. During the sonication process, the liquefied PFB is encapsulated by the PVA chains and a cross-linking acetylation occurs between the aldehyde and hydroxyl groups allowing the formation of a resistant shell (see Figure S1 in the Supporting Information). This process yields a milky white suspension of NDs, which is then left at 5 °C for another hour under mild agitation (200 rpm) prior to washing to continue the cross-linking reaction using a temperature-controlled vortex (Multitherm Shaker) equipped with a block of 15 mL tube holds (from Benchmark Scientific, Sayreville, NJ, USA). 

### 2.2. SizeSorting of PVA/PFB Nanodroplets by Differential Centrifugation

**Figure 1:**
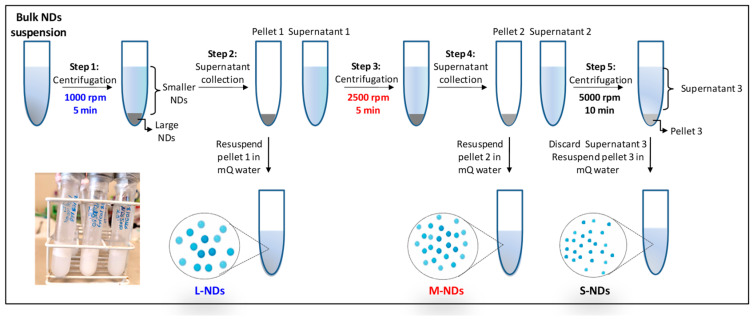
Schematic illustration of differential centrifugation steps for the size sorting of PVA/PFB NDs. The inset picture is a photograph of size-sorted NDs: large (left vial), medium (middle vial), and small (right vial).

Each bulk suspension of PVA/PFB NDs produced at the different sonication durations (i.e., 15 min and 25 min) is size sorted through selective centrifugation steps using a Hettich Universal 320/320R centrifuge (Tuttlingen, Germany) in the following order (See Figure 1):

(1) 1000 rpm (160 g-force, 5 min)
(2) 2500 rpm (1020 g-force, 5 min)
(3) 5000 rpm (4080 g-force, 10 min)

Basically, after the first centrifugation at 1000 rpm, the precipitate (pellet) containing the largest NDs (L-NDs) is separated from the supernatant. The supernatant is carefully pipetted (≈5 mL) without disturbing the pellet and transferred into another centrifuge vial for further centrifugation at 2500 rpm. This step isolates the medium-sized NDs (M-NDs) in the pellet from the smallest NDs remaining dispersed in the supernatant. The supernatant is similarly collected into another centrifuge vial and subjected to a final centrifugation at 5000 rpm. After this final centrifugation, the obtained pellet contains the smallest NDs (S-NDs), while the remaining supernatant, mainly composed of excess PVA chains not involved in the NDs’ encapsulation, is discarded. The precipitates resulting from each centrifugation step are then washed and resuspended in1 mL of mQ water. All size-sorted ND samples are obtained by cumulating preparations from replicated separation processes.

### 2.3. Quantification of Size-Sorted PVA/PFB Nanodroplets

The concentration of the NDs, expressed as a numerical density (NDs/mL), can be estimated by microscopy using a Neubauer counting chamber (0.1 mm × 0.0025 mm^2^). We operated an inverted Eclipse model Ti-E microscope (Nikon Instruments, Tokyo, Japan) equipped with a long-distance 40× objective (S plan Fluor ELWD Ph2 ADM). The lookup table (LUT) was adjusted during image acquisition to improve the contrast and brightness for better visualization of the tiny NDs over the background. The average ND concentration per ml, over time, was calculated on five regions of interest (ROIs) using the Fiji-ImageJ freeware package by adopting the pixel maxima function detecting ND spots.

### 2.4. Dynamic Light Scattering (DLS)

The size (i.e., hydrodynamic diameter) distributions of the various ND samples over time were assessed by DLS using a Malvern instrument (Worcestershire, UK) operating at non-invasive backscatter detection angle of 173° and equipped with a Peltier thermal control and Zetasizer Nano software (v3.30). Each ND sample was typically diluted ×100 in filtered mQ water (using 0.2 μm nylon syringe filters) and filled into plastic disposable cuvettes with 10 mm path length. All measurements were performed at 15 °C to prevent minor spontaneous vaporizations and bubble formation on the cuvette walls. The average diameter of each ND sample was calculated as the mean value of six measurements across three different batches.

### 2.5. Post-Vaporization Size Distribution of Size-Sorted Nanodroplets

We opted for ADV as the activation method to assess the size distribution of microbubbles resulting from the liquid-to-gas transition of the different populations of NDs obtained by size sorting. A volume of 200 μL of each of the ND samples was filled into a microchannel slide (5 mm × 50 mm × 0.4 mm) (Ibidi, Gräfelfing, Germany). Prior to their activation, the ND samples were thermalized for 5 min at 37 °C. ADV activation was achieved using an SP100 sonoporator (1 MHz) (SONIDEL, Dublin, IE) at 100% duty cycle for 30 s and by applying an US power of 2 W/cm^2^. The transition NDs → MBs upon core vaporization was followed by bright field microscopy, before and after the ultrasound exposure, using an inverted Eclipse model Ti-E microscope (Nikon Instruments, Tokyo, Japan) equipped with a 40 × long-working distance objective (S plan Fluor ELWD 40 × Ph2 ADM). The size distributions of the yielded microbubbles were assessed using NIS-Elements AR software (version 4.30) “Annotation and Measurements” tools (Nikon, Milan, Italy).

### 2.6. CarbonIon Irradiation

The irradiation studies of size-sorted PVA/PFB NDs were carried out at the National Center for Oncological Hadrontherapy (CNAO, Pavia, Italy) using a clinical accelerator source producing a C-ion beam with a field size set to 6 × 6 cm^2^. The ND samples were first embedded at the desired concentration into a tissue-mimicking phantom made of a poly(acrylamide) hydrogel matrix contained in ad hoc cuboids, as reported in [17]. Each phantom positioned at the isocenter and immersed in an PMMA water tank (30 × 30 × 30 cm^3^, model 41,023 from PTW) thermalized at 37 °C using a heating homogenizer (accuracy ±1 °C), as depicted in Figure S2a of the Supporting Information. The irradiations were performed on independent phantoms of ND samples under different conditions, i.e., ND concentration, C-ion dose, and beam range (see the summary of irradiation settings in Table S1 of the Supporting Information). The comparison of the response of size-sorted ND samples was evaluated by offline depth-resolved US imaging. The phantoms were scanned before and after exposure to C-ions (with two-ripple filter) using a clinical ultrasound scanner (Mindray DP50, Shenzhen, China) equipped with a linear-array transducer (model 75L38EA, *f_c_* = 7.5 MHz). The probe was fixed with a holding stage and its position was calibrated with the middle of the internal phantom length parallel to the direction of the beam path (acoustic window length = 3 cm). The phantoms were scanned pre-and post-irradiation by taking 3 frames across the full width of the phantom cuboid (See Figure S2b in the Supporting Information). For spread-out Bragg peak (SOBP) and multi-energy irradiations, US images were acquired across the full lateral length by displacing the transducer to cover the entire phantom. For single-spot irradiation, depth-resolved US imaging scans were acquired over the X and Y projections shown in Figure S2b. All frames were obtained using consistent US gain settings (gain value set to 83). A low mechanical index (MI = 0.1) was applied during the scans to prevent acoustic droplet vaporization, which generally occurs at an MI threshold of 0.4.

### 2.7. Imaging Processing and Data Analyses

The pre-and post-irradiation US images were processed using the open-source image processing package “Fiji-Image J” (version v1.53c) without any prior adjustments to brightness or contrast. We used rectangular ROIs to fully select the acoustic window. The ROIs were converted to a 16-bit image and the default threshold profile function was applied to extract the grayscale value contrast maps (i.e., the intensity of pixel brightness). The background signal noise, relative to the contrast of MBs derived from minor spontaneous vaporization in the pre-irradiation images, was subtracted from the post-C-ion exposure profiles. The image coordinates were converted into the effective position within the beam range. The thickness of the cuboid’s wall at the entrance of the beam was taken into consideration to correlate with the exact ND’s vaporization position with respect to the C-ion Bragg peak. All results are presented as the average contrast value corresponding to the 3 frames taken for each phantom along the cuboid width. The measured Bragg peaks corresponding to each range (determined using PeakFinder 2.0 from PTW dosimetry company, Freiburg, Germany) were then compared to the extracted gray-value profiles of the ND US scan. The 50% distal drop in grayscale values was calculated by taking the midpoint between the highest and lowest gray values and used to quantify the range shift of the vaporization profile with respect to the Bragg peak. The shifts in the position of the signals are reported as mean ± standard deviation.

## 3. Results and Discussion

### 3.1. Characterization of Size-Sorted PVA/PFB NDs

**Figure 2:**
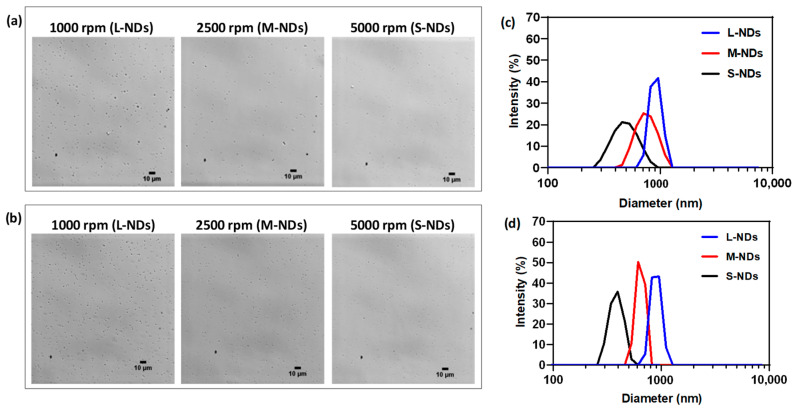
Bright-field microscopy images (objective 40×) of Neubauer counting chambers filled with size-sorted PVA/PFB nanodroplet samples obtained by selective centrifugation and prepared with two sonication durations: (**a**) 15 min of sonication (dilution ×50) and (**b**) 25 min of sonication (dilution ×100). (**c**,**d**) DLS intensity-weighted size distributions of PVA/PFB ND separated populations by selective centrifugation corresponding to (**a**,**b**), respectively. Scale bars: 10 μm.

Differential centrifugation has gained interest in sorting out the distribution of ultrasound contrast agents (UCAs) due to their size-dependent acoustic response [31]. It is generally considered a rapid and affordable approach to size-isolating nano/microparticles of desired distributions without significant sample loss [32,33]. In this context, centrifugation at various speeds exploits selective sedimentation of the NDs based on their size. Bright-field microscopy of the size-sorted PVA/PFB NDs (see Figure 2) shows that, as expected, the NDs collected at lower centrifugation speeds appear larger than those precipitated at higher speeds. Specifically, the largest NDs (L-NDs), collected from the pellet after centrifugation at 1000 rpm, appear as dark spots under the microscope, while the medium-sized NDs (M-NDs) obtained after centrifugation at 2500 rpm are still visible, although smaller. Conversely, the smallest NDs (namely S-NDs), collected after centrifugation at 5000 rpm, appear as very tiny spots, hardly distinguishable from the background of the microscopy image. It is worth noting that the concentration of the smallest population of NDs is likely underestimated, as their size falls at the microscope’s resolution limit (≈400 nm), even when labeled with a fluorescent dye (Rodhamine B isothiocyanate, RBITC) (see Figure S3 in the Supporting Information). This first qualitative evaluation demonstrates a successful selective sorting of ND populations by size. The described preparation method typically yields an ND concentration ranging between 10^9^ and 10^10^ NDs/mL, with no striking differences between the various size-sorted populations. However, the concentration outcome of the ND preparation process is an indicative estimation and can vary due to experimental handling factors such as the amount of condensed gas and the temperature of the water bath sonicator. As a routine analysis, the concentration is quantified for each freshly prepared sample.

Figure 2c,d show the intensity-weighted size distributions of the separated populations of PVA/PFB NDs obtained from the bulk preparations produced by sonication for 15 and 25 min. With respect to the original formulation protocol, where the sonication process lasts for 15 min, extending the sonication time is believed to primarily impact the shell thickness and the cross-linking density rather than the average size. Notably, increasing the centrifugation speed (from 1000 to 5000 rpm) results in a decrease in the peak and upper cutoff of the ND size distributions. The shift in NDs’ size diameters is more pronounced when comparing L-NDs and S-NDs. While the size distribution of the medium populations (M-NDs) overlaps with both the larger (L-NDs) and smaller (S-NDs) populations. However, in the samples sonicated for 25 min (Figure 2c) a better separation is observed between the three size distributions. 

Table S2 in the Supporting Information summarizes the mean diameter of size-sorted NDs obtained with the two sonication processes. Differential centrifugation led to average diameters of approximately 400 nm, 650 nm, and 900 nm, respectively, for S-NDs, M-NDs, and L-NDs collected from the various samples. The centrifugal separation relies on the density difference between the superheated liquid PFB core (≈1.517 g/mL) and the aqueous medium. However, the sedimentation speed is determined by the NDs’ hydrodynamic diameters and this factor can be useful for a size-sorting process. The larger PVA/PFB NDs (i.e., encapsulating higher volume of PFB) settle preferentially at a lower centrifugation speed or spontaneously within a shorter time to form a pellet. However, smaller NDs remain in the supernatant and require a higher centrifugation speed or a longer time to sediment. While microfluidics or extrusion technologies offer better size control of droplets, they necessitate specialized custom equipment and have limited throughput. Moreover, to generate specific size ranges of NDs, these methods are typically integrated into the encapsulation process and are mostly suitable for higher b.p. perfluorocarbons that are liquid at RT. One could also argue that filtration can potentially separate NDs instead of centrifugation, but the process is susceptible to filter clogging, low throughput, and significant loss or damage of the sample.

### 3.2. Shelf-Life Stability of Size-Sorted NDs

**Figure 3:**
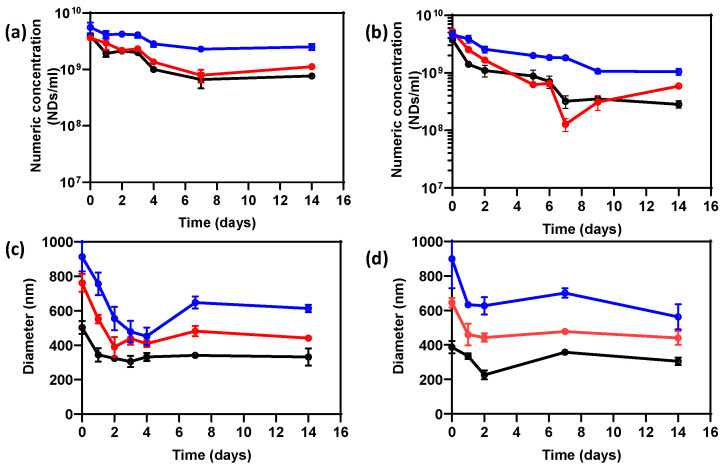
Concentration variation over time of size-sorted PVA/PFB NDs, i.e., L-NDs (blue), M-NDs (red), and S-NDs (black) produced by (**a**) 15 min sonication and (**b**) 25 min sonication. (**c**,**d**) Corresponding mean diameter variation over time of the size-sorted NDs produced by 15 min and 25 min sonication, respectively. The lines are a guide for the eye.

The shelf-life stability of the various size-sorted PVA/PFB ND samples was investigated by monitoring changes in both the numeric concentration and size of the NDs over a two-week period. This timeframe was chosen based on previous experiments where no significant changes were observed after 14 days. Microscopy (as described in Section 2.1) and DLS measurements were used for this purpose. The cross-linked PVA thick shell allows for enhanced ND stability against coalescence, though we do not exclude that NDs are prone to external stimuli altering their stability during storage and handling. Figure 3a,b illustrate the variations in concentration for the L-NDs, M-NDs, and S-NDs populations obtained with the different sonication times (i.e., 15 and 25 min). We notice that the concentration tends to decrease more remarkably for all the samples over the first week, then remains more stable. Some fluctuations, such as those seen in M-NDs (Figure 2b), can be attributed to experimental uncertainties in the counting method of NDs. The numeric concentration of S-NDs seems to decrease slightly more, but this behavior could be related to an underestimation, as the tiny NDs below 450 nm are difficult to visualize given the limitations of the microscope resolution. This suggests that their effective concentration may be higher than the calculated value. 

The decrease in ND concentration over time of is a common behavior also reported for high-boiling-point PFC-based NDs, such as perfluoropentane (b.p. = 29 °C) and perfluorohexane (b.p. = 56 °C), and is hypothesized to be caused by leakage of PFC from the NDs. The concentration loss is usually more pronounced in lower b.p. NDs due to their reduced core stability [34]. 

On the other hand, we observe a decrease in the NDs’ average size for most samples over the first 4 days (Figure 3c,d), followed by a stabilization or even an enlargement (e.g., L-NDs Figure 3c). The initial decrease in the NDs’ mean size may be due to the spontaneous vaporization of a few NDs present in each sample that are less affected by the Laplace pressure exerted by the shell [23]. This behavior is consistent with the observed decrease in numeric concentrations. However, the size growth after a few more days in some cases can be attributed to the swelling of some NDs as a result of partial vaporization of the PFB metastable core. 

### 3.3. Size Expansion Evaluation of Size-Sorted NDs by ADV

**Figure 4:**
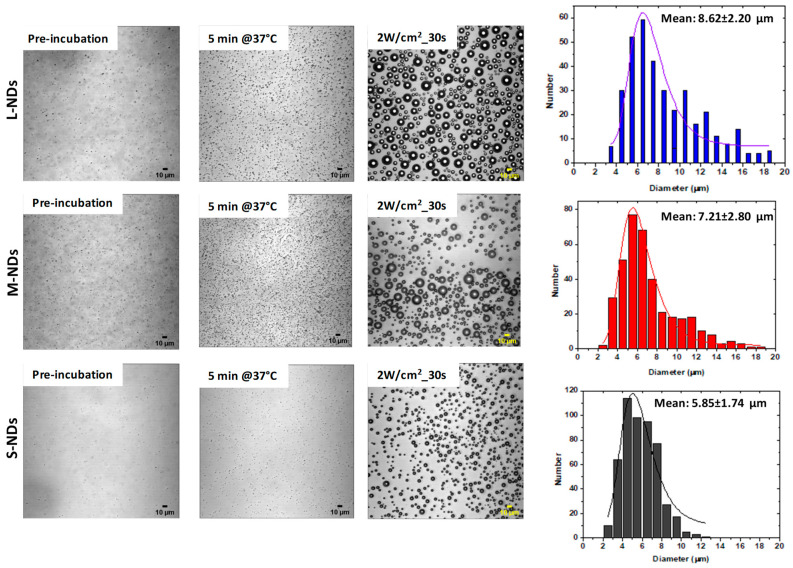
ADV study of size-sorted PVA/PFB NDs assessed by bright-field microscopy (long-distance objective 40×) in ibidi µ-channel slides. We show the ND images upon channel loading (pre-incubation), after thermalization at 37 °C, and after acoustic activation using 1 MHz US transducer (Sonidel, 2 W/cm^2^, 30 s). On the right side we show the size distributions of the generated microbubbles upon phase-transition, corresponding to each size-sorted ND sample. The ND samples were produced after 25 min sonication. Scale bars: 10 μm.

The vaporization of phase-change NDs into microbubbles requires energy to overcome the nucleation barrier within the core, conventionally achieved acoustically by applying appropriate insonation power. The occurrence of liquid-to-gas transition that differentiates MBs from NDs is observable in microscopy. This transition is marked by an abrupt, irreversible size increase from the submicron to the micron range, with MBs appearing as dark rings due to the refractive index difference at the gas–liquid interface. The size of the MBs generated from vaporized NDs could be influenced by several factors, including gas uptake from the host medium into the MBs, initial size, and the core composition [35]. Figure 4 shows the vaporization of size-sorted PVA/PFB NDs @37 °C upon acoustic activation using a sonoporator (1 MHz, 2 W/cm^2^, 30 s, duty cycle 100%), assessed by microscopy in order to determine the obtained size distribution after NDs are converted to MBs. Before activation, L-NDs and M-NDs are more clearly visible than S-NDs, even after incubation at 37 °C, where swelling (enlargement) of the NDs was observed for all samples. Interestingly, while all size-sorted NDs could be activated under the applied US power, there is a striking difference in the size distributions, in particular when comparing L-NDs and S-NDs. M-NDs appear to lead to the less uniform size range of MBs, exhibiting a mix of large and tiny MBs; this is expected since their size distribution obtained by DLS overlays with the larger and tinier populations. In correlation with the average initial size of the NDs, the mean diameters of the resulting MBs are approximately 8, 7, and 5 µm for L-NDs (1000 rpm), M-NDs (2500 rpm), and S-NDs (5000 rpm), respectively; in other words indicating that smaller NDs lead to smaller MBs. This experiment suggests that even the largest population of NDs produce MBs with adequate average dimensions under 10 µm, suitable for intravenous injection into blood capillaries. It is worth mentioning that no significant difference was observed between the size-sorted NDs produced after 15 min or 25 min sonication. However, we believe that some difference could exist in the µs time-scale during the ADV process that becomes negligible as the MBs experience characteristic time-dependent growth due to influx of dissolved gases present in the surrounding medium [36]. 

### 3.4. CarbonIon Response of Size-Sorted PVA/PFB NDs

Our previous study confirmed the feasibility of this proof of concept using bulk suspensions of NDs, focusing only on their response to beam settings such as dose and intensity, without accounting for the influence of ND size [17]. The PVA/PFB NDs were primarily activated by C-ion radiation at the Bragg peak, where charged particles achieve their maximum LET, thus accomplishing the activation threshold of the NDs’ superheated cores. The calculated LET required to vaporize metastable PFB is 145 keV/μm at 37 °C [16]. This LET threshold varies with the degree of superheat and, consequently, with temperature. The large NDs exhibit slightly lower superheating limit temperatures (i.e., inducing their spontaneous vaporization) compared to the smaller sub-populations, as measured by the DSC (see Figure S4 in the Supporting Information). It is well known that the stability of NDs against activation stimuli increases as their size decreases, because smaller NDs experience higher Laplace pressure [37]. However, we did not evaluate the temperature effect when examining the response of size-sorted NDs to C-ions. All irradiations were performed at physiological body temperature considering that the NDs are designed for in vivo dosimetry. Additionally, given that medium-sized NDs exhibited a heterogeneous size distribution when converted to MBs, appearing as a mixture of the largest and smallest populations, and due to beam availability limitations, we focused irradiations mainly on L-NDs and S-NDs to assess the differences related to ND properties and/or beam settings for C-ion response detection.

#### 3.4.1. The PVA/PFB NDs C-Ion Radiation-Sensitivity Depends on the Size

**Figure 5:**
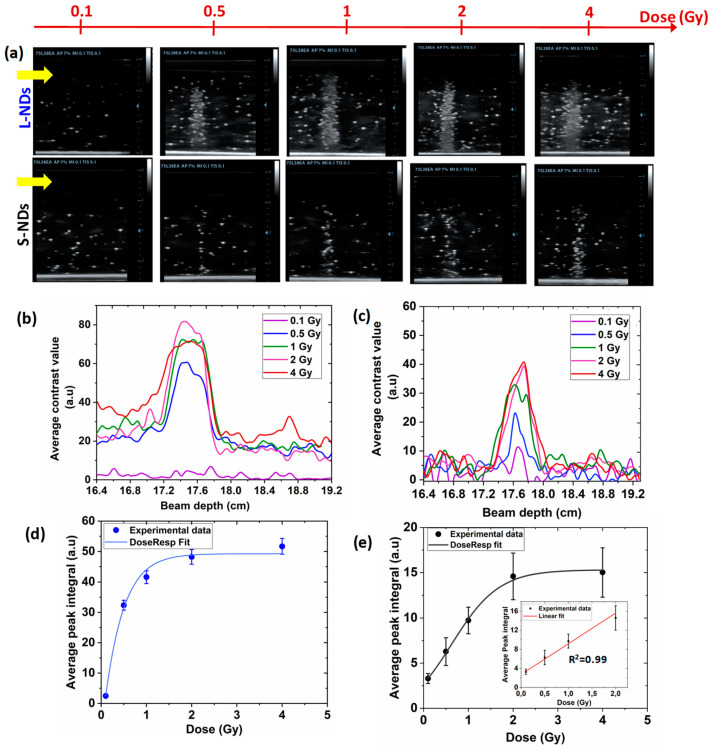
Comparison of the size-sorted ND (25 min sonication) response to C-ions (312 MeV/u, 180 mm range) at 37 °C after exposure to doses between 0.1 and 4 Gy: (**a**) depth-resolved US images (7.5 MHz, MI = 0.1) of independent phantoms of NDs dispersed in PAM (8.4 × 10^5^ NDs/mL) post-irradiation at the different tested doses (the yellow arrows indicate the C-ions beam’ entrance side). (**b**,**c**) Average contrast gray-value profiles from the vaporization of L-NDs and S-NDs, respectively, at the various doses (number of frames/phantom = 3). (**d**,**e**) Evaluation of the peaks’ integrals from the average grayscale profiles of L-NDs and S-NDs, respectively, as a function of C-ion dose. The inset shows the linear regression fit up to 2 Gy.

Figure 5 illustrates the influence of the C-ion dose on the induced vaporization intensity of both L-NDs (≈900 nm) and S-NDs (≈400 nm). Examples of US images of the phantoms prior the C-ion exposures are shown in Figure S5a of the Supporting Information. When in the liquid state, the core of NDs shows no signal in US imaging; contrast is only generated when the NDs are converted to MBs, inducing an acoustic mismatch with the surrounding tissue. To this aim, the few bright spots observed in the phantoms before irradiation are considered a noise background resulting from minor spontaneous vaporization of the NDs during the phantom preparation. The ND concentration in the phantoms was the same for both samples (i.e., ≈10^6^ NDs/mL). These NDs were size sorted from the same bulk preparation, obtained with 25 min sonication. It is clear that in the two cases, the response of NDs depends on the dose of C-ion radiation. The US images of different phantoms exposed to C-ions from 0.1 to 4 Gy show a gradual increase in the contrast, which is attributed to the generation of MBs upon the phase transition of NDs at the distal position corresponding to the BP (Figure 5a). Nevertheless, as we compare L-NDs and S-NDs at each C-ion dose, and considering irradiations were performed at the same C-ions range of 180 mm (312 MeV/u), the large population exhibits evidently higher and saturated echo-contrast due to the multiple scattering from MBs compared to the small NDs that, contrarily, show a lower signal within a narrower range, suggesting the occurrence of fewer vaporization events. We previously reported that by analyzing and plotting the lateral grayscale value profiles derived from the US images (see paragraph 2.7 in the Materials and Methods section), the vaporization of bulk NDs at the Bragg peak, exposed to the same energy of C-ions, can be assimilated to a Gaussian peak. This finding is further confirmed herein for S-NDs and for the L-NDs below 1 Gy, as depicted in Figure 5b,c. Above such dose the large population seems to follow a similar trend to the Bragg peak at the plateau, i.e., before the distal fall-off, (see Figure S5b at the Supporting Information). The contrast enhancement in the plateau is attributed to the vaporization of a portion of NDs before the Bragg peak, hence suggesting that larger NDs could require a lower LET to trigger their activation. Additionally, the quantified grayscale values for S-NDs at the high doses are significantly different; about three times lower than for L-NDs (Figure 5d,e). The influence of droplet diameter has been studied for larger populations of the same formulation in the micron range between 2 and 6 μm and being irradiated with a proton beam at 50 °C [38]. The authors observed an increase in the vaporization response with the droplet size, showing a similar trend to the theoretical estimates derived from the thermal spike theory [39,40]. In agreement with these findings, we confirm that even when the LET threshold predictions are met, vaporization events as a response to C-ion radiation occur less for smaller NDs in the sub-micron range. 

The measurement of the peak integrals of the generated grayscale contrast for each C-ion dose in both ND populations can be fitted with a dose–response or exponential function (in Origin Pro 8.0 software). The US contrast increases linearly (R^2^ = 0.99) at low doses up to 2 Gy for the S-NDs population, after which it reaches a plateau. It is known that extensive ND activation increases acoustic attenuation, thereby hindering the detection of new vaporization events. However, it is evident that for S-NDs the plateau is not due to the saturation of the US signal as observed with L-NDs. Instead, it is more likely due to the low probability of vaporization events, as the contrast enhancement stripe in the US images remains clear even at the high dose of 4 Gy. As a matter of fact, the phase transition of NDs is driven by the formation and collapse of vapor embryos in the core, creating regions with liquid–vapor interfaces inside the droplet. These gas pockets keep expanding until they reach a critical radius when the radiation exposure becomes sufficient to nucleate an adequate number of embryos [24]. As the formation of vapor embryos is expected to be less probable in smaller NDs, this could explain the lower vaporization yield observed.

#### 3.4.2. The NDs’ Radiation-Sensitivity Depends on the Concentration

We investigated the influence of ND concentration on the vaporization yield detected by US imaging after exposure to a minimal dose of C-ions, specifically 0.1 Gy (312 MeV/u). Primarily, we used the large-sized sub-population of L-NDs obtained with 25 min of sonication and measured the contrast enhancement as a function of ND concentration in the phantom, ranging from 8.4 × 10^5^ to 4 × 10⁶ NDs/mL. At the lowest tested concentration, no response was detected. However, as we increased the ND concentration to 2 × 10⁶ NDs/mL, significant contrast enhancement was observed at the beam depth relative to the BP. Further increasing the concentration by a factor of two resulted in a proportional enhancement of the contrast signals at the BP due to the increased number of vaporization events (see US images in Figure S6a in the Supporting Information). The response of the NDs (Figure S6b) was linearly related to their concentration in the phantom at 0.1 Gy, exhibiting a regression coefficient of R^2^ = 0.99.

Interestingly, an excellent correlation was also found when comparing NDs’ responses with variations in both concentration and dose by opposite factors. Specifically, irradiating L-NDs with 0.5 Gy C-ions (312 MeV/u) at a concentration of 8.4 × 10^5^ NDs/mL resulted in the same contrast enhancement as when the ND concentration in the phantom was approximately five times higher (i.e., 4 × 10⁶ NDs/mL) but exposed to 0.1 Gy (312 MeV/u), as illustrated in Figure S6c. However, we point out that the correlation between ND concentration and dose may only be valid at low doses of C-ions, because at high doses massive vaporization occurs, leading to the saturation of acoustic signals. In addition, S-NDs exhibited an US contrast enhancement at 0.1 Gy when their concentration was doubled to approximately 2 × 10^6^ NDs/mL, but the signal was evidently lower than L-NDs (Figure S6d in the Supporting Information), confirming that smaller NDs require a higher concentration to give a visible signal than larger ones.

It is worth mentioning that the tested concentrations in the phantoms giving strong echo-contrast signals are considerably low and well below the expected safely-tolerated doses. We reported that the bulk suspension of PVA/PFB NDs shows good in vitro biocompatibility up to a concentration of 2 × 10^8^ NDs/mL, with cell viability above 80%, while at higher concentrations the NDs are believed to be non-toxic and only act as a barrier to cells, inhibiting their proliferation since they tend to sediment and form a thick layer over the cells due to the high density of the PFB core [12]. Indeed, the reported typical concentrations used in vitro for the US detection of activated NDs are up to 5 × 10^6^/mL [41]. In tissue-mimicking phantoms, as the NDs are entrapped and the vaporization leaves voids leading to high echogenicity and easily saturation, while in vivo, the threshold of detection and saturation could be higher as the NDs are circulating and prone to diffusion and washout.

#### 3.4.3. The NDs’ Radiation-Sensitivity Depends on the C-Ion Range

**Figure 6:**
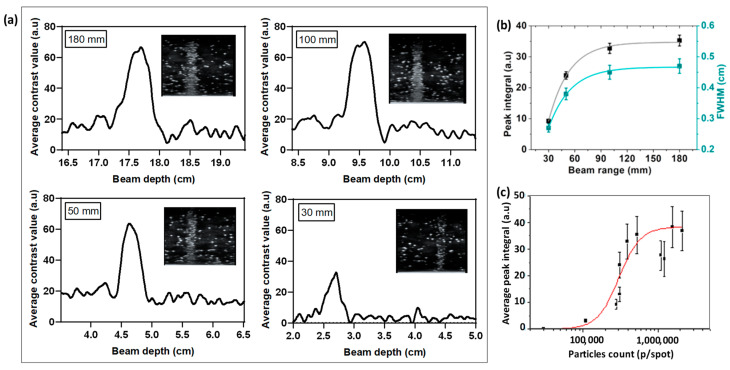
(**a**) Average contrast gray-value vaporization profiles extracted from US images (inset figures, MI = 0.1, 7.5 MHz) of S-NDs (obtained after 15 min sonication) post1Gy C-ion irradiation with different ranges between 30 and 180 mm at 37 °C (the pre-irradiation background profile was subtracted). The ND concentration in the phantoms is 10^6^ NDs/mL. (**b**) Corresponding evaluation of both vaporization peaks integrals and FWHM from the average grayscale profiles of the NDs as a function of the C-ion range (the curves are fitted with an exponential function). (**c**) Summary plot of measurements of echo-contrast peak integrals of S-NDs (15 min sonication, C ≈ 10^6^ NDs/mL) irradiated under different doses/ranges as a function of C-ion particle count/spot (the red line is a logistic fit).

We noticed that the vaporization yield of NDs in the Bragg peak depends on the C-ion range. When comparing the response of phantoms made from the same sample of NDs at the same concentration and being exposed to the same dose of C-ions at various ranges, we found an increase in the contrast enhancement with the range of C-ions. Figure 6a depicts post1Gy US images of S-NDs (obtained with 15 min sonication) and the corresponding average gray-value vaporization profiles resulting from exposure to C-ions with ranges of 30 mm, 50 mm, 100 mm, and 180 mm. The ND response at 30 mm is significantly lower compared to higher depths. The measurements of the generated gray-value echo-contrast and the FWHM of the ND vaporization peaks show exponential growth as a function of the range (see Figure 6b and Figure S7a). 

The broadening of NDs’ vaporization peaks with the increase in beam depth is expected, as it is well known that the width of the BP in heavy particles, including C-ions, tends to increase for longer ranges (i.e., high energies) due to the spread of energy depositions becoming more elevated as the charged particles travel through more material/tissue, leading to extensive scattering and broadening [42,43]. The comparison of the widths of the vaporization peaks and the measured broadness of the BPs’ at W_80_ (i.e., the width of the peak at 80% of its maximal dose), summarized in Table 1, shows an excellent match with sub-millimeter errors, except for the 30 mm range where the ND vaporization peak is approximately twice as narrow as the BP. This suggests that at 30 mm, a slightly higher concentration of S-NDs would be needed to improve the spatial distribution precision. 

One could argue that heavy particles with shorter ranges are characterized by a higher LET because they typically start with less energy and lose it more rapidly over a shorter distance, hence inducing higher energy deposition. However, we observed a decay of the NDs’ responses at shorter ranges. The sensitivity of NDs with a specific superheating degree (as described by Equation (2)) to ionizing radiation is definitely linked to the LET. The LET threshold required for PFB-based NDs is estimated to be 145 keV/μm, which is below the maximum achieved by C-ions [27]. However, it appears that when this threshold is exceeded the mechanism behind the NDs’ vaporization efficiency remains complex to define, as it is influenced by several factors affecting the ND sensitivity. 

The plot of NDs’ responses as a function of the C-ion particles/spot (p/spot) corresponding to each range after 1 Gy of exposure shows similar exponential growth (Figure S7b of the Supporting Information). We assume that the ND vaporization yield could be related to the fluence of the C-ions, as the ND response is also dose dependent and increasing the dose for the same range requires increasing the number of particles/spot, as reported in Table S1 of the Supporting Information. We hypothesize that higher fluence increases the ionization probability, as more C-ion particles are likely to interact with the NDs as they slow before stopping. This assumption could also explain why a higher concentration of NDs is needed at low doses (below, e.g., 0.1 Gy) to be detected. To further support this hypothesis, we summarized the response of the same batch of S-NDs at the same concentration as a function of the number of particles/spot set for various ranges/doses. Although the plot presents some fluctuations (see Figure 6c), it shows a clear increase in the quantified US signals with C-ion particle counts up to ≈10^6^ p/spot.

#### 3.4.4. The NDs’ Radiation-Sensitivity Depends on the Shell Properties

**Figure 7:**
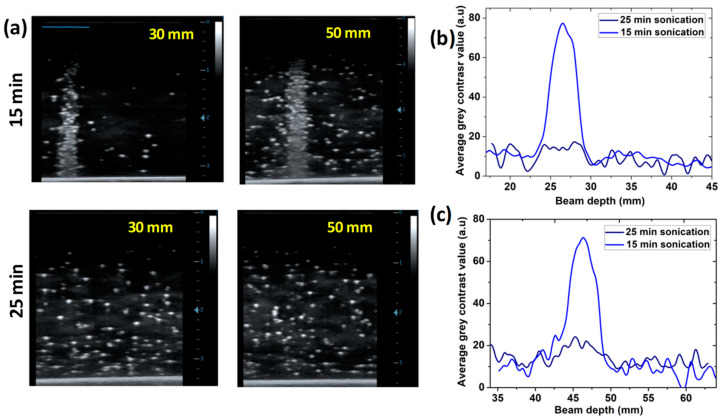
(**a**) Comparison of US images (MI = 0.1; 7.5 MHz) of L-NDs (C ≈ 10^6^ NDs/mL), obtained with sonication durations of 15 min and 25 min, after irradiation with 1 Gy C-ions at 30 mm and 50 mm ranges. (**b**,**c**) Comparison of the gray-value vaporization profiles of L-NDs obtained at the different sonication durations corresponding to the exposure to C-ions with ranges of 30 mm and 50 mm, respectively. The pre-irradiation background was subtracted from the profiles.

So far, the sensitivity of NDs to C-ions depends on their concentration, size, and particle count linked to beam range or dose, but irradiation experiments have shown that the shell properties also play a crucial role in influencing their response yield. NDs produced after 25 min of sonication and exposed to 1 Gy of C-ions at a concentration of ≈10^6^ NDs/mL showed a response only for 180 mm and 100 mm. For the tested ranges below (i.e., 50 mm and 30 mm), at the same irradiation dose, a higher concentration was required in the phantom to observe evident vaporization of the NDs at the BP. However, without increasing the concentration, the ND formulations produced with a shorter sonication time (15 min) showed a clear response when irradiated with the lower energies of C-ions. Figure 7 illustrates the comparison between the L-NDs produced with 15 and 25 min of sonication after 1 Gy of C-ions at 30 mm and 50 mm. The vaporization of the NDs is barely detected for the sample with longer sonication time, exhibiting low contrast enhancement at the BP with a high signal-to-noise ratio. In contrast, the sample obtained after 15 min of sonication exhibited a strong response, leading to a sharp peak for both ranges. This behavior is also confirmed for S-NDs, where a clear response is observed at the low-path ranges with the formulation obtained after 15 min sonication, as shown in Figure 6. 

Although no significant differences were observed in the shelf-life stability of the formulations sonicated for different lengths of time, C-ion-triggered vaporization events occurred less for samples produced after 25 min of sonication. These NDs seem to experience more resistance to ionizing radiation, likely due to an increased number of cross-links of the PVA shell or more PVA chains being deposited on the PFB drop interfaces, making the shell more robust or thicker [44,45]. It has been reported that the typical activation dynamics of perfluorocarbon NDs, either optically or acoustically, can vary drastically depending on their composition and coating properties. For example, Welch et al. noted that, for lipid-shelled NDs, adding too much PEGylated lipid could create steric hindrance and make the shell too stiff for efficient expansion, thus lowering the acoustic signal after activation [46]. Similarly, the expansion of NDs activated by the ionizing radiation could be affected by the shell thickness and visco-elastic properties. Therefore, the results suggest that increasing the sonication time is not advantageous for our purpose, as the NDs obtained after 15 min show good response to C-ions at lower concentrations in addition to a similar shelf-life stability.

#### 3.4.5. Dose-Linearity Dependence of NDs to C-Ions

In previous research, we reported on the dose dependence of polydisperse PVA/PFB ND bulk suspension to C-ions without considering the range [17]. Figure 5d,e confirmed our previous findings, showing dose linearity up to 2 Gy after exposure of S-NDs and L-NDs to 180 mm C-ions beam (≈312 MeV/u). By evaluating the dose linearity of the echo-contrast enhancement from the vaporized NDs at a 50 mm range (≈151 MeV/u), we noticed an improvement in linearity for both size-sorted NDs up to 4 Gy, with a squared correlation coefficient R^2^ = 0.99 (see Figure S8a,b in the Supporting Information). The expanded linearity interval is due to the lower response of NDs, which prevented saturation or acoustic shadowing in the US images caused by the multiple scattering of the generated MB cloud. We believe that increasing the dose at 50 mm above 4 Gy would similarly result in signal saturation. However, we limited our investigation to the maximal doses that are used in clinical practice for one irradiation fraction [47].

#### 3.4.6. Selective Response of NDs to the Beam Settings for Accurate Range Verification

As mentioned above, range measurements in hadron beams are very important to detect deviations, because a small shift in the range can lead to a significant and undesired change in dose distribution. Verification methods can involve a single BP or more sophisticated approaches. The single BP irradiations (shown in Figure 6c and Figures S5b and S8c–e in the Supporting Information) clearly demonstrate the potential of PVA/PFB NDs for range verification. Most irradiation measurements, regardless of the ND size, resulted in sub-millimeter accuracy in terms of the R50 distal fall-off of the BP (the depth at which the dose has dropped to 50% of its peak value) and W_80_ broadness (see Table S3 in the Supporting Information). A few exceptions requiring higher concentrations to gain better spatial distribution of the generated US signal were observed, particularly with S-NDs at low ranges/doses, where the vaporization profiles are narrower compared to the BP. In contrast, L-NDs at doses higher than 2 Gy produce broad vaporization profiles. It is worth mentioning that the selectivity of the NDs’ responses specifically at the BP can be explained by the fact that at the plateau (before the BP), the energy deposition of C-ions is low because the particles travel faster, resulting in a lower probability of interaction with the NDs in phantom. While at the BP, the C-ions achieve their maximal LET because they slow down and release all their energy before stopping and are likely to interact more with the traveled material. Nevertheless, a slight contrast enhancement occurring in the plateau following the trend of the BP curve was observed for doses above 1 Gy. This enhancement is more pronounced in L-NDs samples (see Figures S5b and S8d), indicating the vaporization of a small fraction of NDs before the BP triggered by lower LET values.

We further investigated the response of size-sorted NDs under more complex irradiation settings to verify how well the generated US contrast maps from vaporized droplets match with the beam range parameters. Indeed, in clinical practice, radiation treatment is tailored based on the tumor characteristics and patient-specific factors. To this end, we opted for various settings including spread-out Bragg peak (SOBP), multi-paint irradiation, and single spot. 

**Figure 8:**
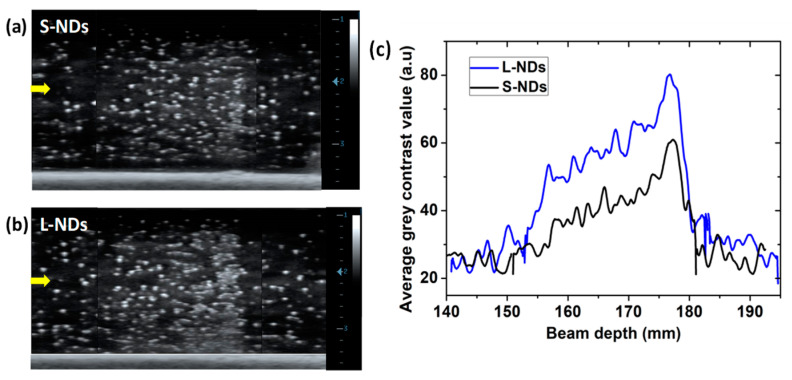
(**a**,**b**) US image (MI 0.1, 7.5 MHz) of S-NDs and L-NDs post 1 Gy SOBP C-ions (160–180 mm) and (**c**) corresponding average gray contrast-value profile corresponding to generated MBs. Both NDs samples were produced with 15 min of sonication. The concentration of L-NDs and S-NDs in the phantoms is C ≈ 10^6^ NDs/mL. The yellow arrows indicate the beam entrance side.

SOBP irradiation is commonly used to cover an entire tumor volume, as a tumor might extend at varying depths and a single BP is not sufficient for effective and precise treatment. The SOBP is created by stacking multiple BPs at different depths and weighted energies, forming a continuous dose-deposition region. Herein, S-NDs and L-NDs were exposed to 1 Gy SOBP in the range of 160–180 mm. US imaging scans of the full lateral length of the phantoms, shown in Figure 8a,b, reveal contrast enhancement over a wide range. The corresponding average gray-value profiles (Figure 8c) show a distal fall-off of both samples at 180 mm, with vaporization starting approximately at 155 mm, consistent with the first BP depth expected at 160 mm. In agreement with the previous results, S-NDs at the same concentration as L-NDs exhibit a lower vaporization yield and hence a lower US signal. However, by doubling the S-NDs concentration to 2 × 10^6^ NDs/mL (see Figure S8e in the Supporting Information), the contrast generation in the SOBP range becomes clearly intense. We observed that the contrast generation gradually increases until the end of the SOBP, where the ND vaporization yield is maximal. This behavior was observed previously in bulk ND suspension and is confirmed herein. In therapeutic practice, SOBP is designed to ensure a uniform dose across the irradiated target volume so that the entire tumor receives a therapeutic dose [48]. Therefore, we believe that the gradual increase in vaporized NDs is not due to a gradual increase in the C-ion dose but is rather linked to the range and variation of the incident particle count in the cumulative BPs (See Table S1 in the Supporting Information).

**Figure 9:**
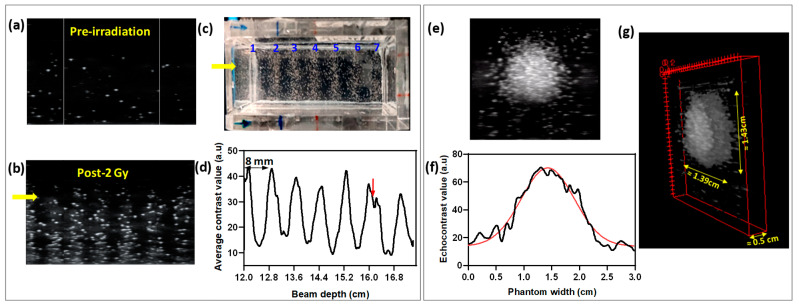
(**a**,**b**) US images of L-NDs (25 min, C ≈ 10^6^ NDs/mL) pre and post2Gy multi-energy C-ions irradiation(i.e., 7 energies from 126 to 174 mm with energy step of 8 mm). The yellow arrows indicate the beam entrance side; (**c**) top-view photograph of the irradiated phantom and (**d**) corresponding contrast gray-value vaporization profile of NDs (the red arrow in d is an imaging artifact caused by the combination of images to cover the full length of the phantom). (**e**) US image of S-NDs (15 min, C ≈ 10^6^ NDs/mL) post 5 Gy single spot C-ion irradiation with an energy of 399 MeV/u (the US transducer is perpendicular to the beam direction); (**f**) corresponding contrast gray-value profile (the red line is a Gaussian fit) and (**g**) 3D US map reconstructed from a full scan of the spot with two projections (i.e., perform scanning with the transducer perpendicular and parallel to the beam direction). The C-ion total count in the spot is 8.2 × 10^6^.Pre-irradiation profiles were subtracted.

Second, we performed multiple paint C-ion irradiation to further confirm how NDs can be selectively vaporized at the BP, excluding any non-homogeneity in their distribution in the phantom. This process involves delivering the C-ions in multiple passes over the full lateral length of the phantom. We delivered seven Bragg peaks, each separated by 8 mm at depths ranging from 126 mm to 174 mm. Figure 9a–d show the outcome for an L-NDs phantom exposed to 2 Gy multi-paint C-ions. Consistently with the beam settings, the phantom after irradiation presents seven visible stripes with generated bubble clouds (Figure 9c). US imaging scans over the full length of the phantom parallel to the beam entrance reveal the presence of seven contrast-enhancement peaks. Each of the peaks in the extracted gray-value profile can be fitted with a Gaussian function (Figure S9 in the Supporting Information), all resulting in similar areas and widths. This similarity is expected as the number of C-ion particles per spot was fixed to 8.2 × 10^6^ for all energies. The average measured peak-to-peak distance (quantified either at the center or the fall-off of each peak) in the extracted gray-value profile is 8.2 ± 0.7 mm. These results highlight the excellent match between the response of NDs and beam settings, demonstrating their potential for highly precise range verification. 

Furthermore, range verification in C-ion beams can also be approached by focusing on individual spots. Spot scanning allows for highly adaptable dose distributions capable of conforming closely to complex and irregular tumor shapes, thereby providing better control over anatomical changes during treatment. However, this approach presents increased complexity and necessitates sophisticated tracking systems. In a water-equivalent medium, the lateral spot spreading is described by a 2D Gaussian for pencil beams [49,50]. We exposed a phantom of S-NDs to 5 Gy distal spot C-ions (i.e., closest to the maximum penetration depth of the beam) with an approximate energy of 400 MeV/u. We analyzed the vaporized NDs triggered by C-ions to verify the shape and spatial distribution of the spot. US imaging scans of the irradiated phantom were performed perpendicularly and parallel to the beam entrance, as shown in Figure S10 of the Supporting Information. The spot thickness measured from the longitudinal profiles obtained by the parallel scan is 5 mm. Figure 9e illustrates the US image of the spot taken perpendicular to the beam, which was used to evaluate the lateral profile of the spot. The extracted contrast-enhancement profile, attributed to the S-NDs vaporization in Figure 9f, reveals, as expected, a Gaussian distribution with FWHM = 11.25 mm and σ = 4.78 mm. Moreover, by stacking images from the US scan of the NDs’ phantom in the two projections, we can reconstruct a 3D map of the C-ion spot, as shown in Figure 9g. These results validate the NDs’ efficiency for more complex range verification, especially considering that distal spots are the most critical in the uncertainties measurement for heavy particle therapies [18,19,20]. The potential of US imaging to detect the ND response could be a reasonable and non-invasive solution to provide precise range verification and effective monitoring of deviations. 

## 4. Conclusions

Differential centrifugation was used to size isolate PVA/PFB NDs from the bulk emulsions produced by sonication for either 15 or 25 min. Experiments on S-NDs (≈400 nm) and L-NDs (≈900 nm) revealed that all samples showed good sensitivity to C-ions even at a low dose of 0.1 Gy, irrespective of size or sonication time but depending on the ND concentration. However, under identical conditions, the vaporization yield of NDs triggered by C-ions is affected by their properties. The generated US contrast signal from the vaporized NDs increases with the C-ion fluence, which explains the higher response of NDs at elevated doses and higher beam energies. Consequently, the size-sorted NDs demonstrated better dose linearity up to 4 Gy at a 50 mm range (151 MeV/u) compared to up to 2 Gy at a 180 mm range (312 MeV/u). The vaporization of NDs is primarily triggered at the BP with sub-millimeter precision. Both L-NDs and S-NDs demonstrate promising potential for C-ion range verification, not only for single BP irradiation but also in response to complex beam settings such as SOBP, multi-paint, or distal spot. However, when the C-ion fluence is very low or very high, the spatial distribution of the generated echo-contrast should be optimized for the size-sorted NDs by adjusting their concentration. It is worth mentioning that the highest concentrations of NDs tested in the phantoms were shown to be biocompatible in other studies, suggesting the suitability of the use of NDs for intravenous injection. Longer sonication times (>15 min) in the ND formulation process are not beneficial as they could affect the shell’s visco-elastic properties, lowering the ND vaporization yield from C-ions and requiring higher concentrations. 

Overall, the results highlight the potential of PVA/PFB nanodroplets for C-ion dosimetry and range verification. Additionally, the good response of the S-NDs population with an average size of 400 nm is important from a clinical perspective since their small size could facilitate passive targeting and uptake by tumor tissues. Ongoing experiments aim to confirm these findings by evaluating the behavior and response of size-sorted NDs with cell cultures. 

One could argue that in vivo diffusion and washout may limit the dosimetric performance of the NDs and their spatial precision for range verification. We believe that adopting online high-frame US imaging, rather than the offline modality used in this work, could counteract any of these potential limitations and track in real-time the echo-contrast signals resulting from the phase transition of NDs. 

## Figures and Tables

**Table 1:** Analyses of the S-NDs vaporization profiles post1Gy at different ranges vs. the measured Bragg peak data from PeakFinder. The results derive from a Gaussian fit of the vaporized ND peaks. NA means not applicable.

Energy (MeV/u)	311.85		221.45		150.71		115.23	
Range (mm)	180		100		50		30	
Particle Count/Spot	5.3 × 10^5^		3.9 × 10^5^		3.1 × 10^5^		2.8 × 10^5^	
	NDs	BP	NDs	BP	NDs	BP	NDs	BP
**Area (a.u)**	35.25 ± 1.1	NA	32.73 ± 1.3	NA	23.97 ± 1.2	NA	9.23 ± 0.4	NA
**x_c_ (cm)**	17.65 ± 0.01	17.69	9.58 ± 0.02	9.68	4.61 ± 0.01	4.68	2.71 ± 0.04	2.68
**x_end_ (cm)**	18.07 ± 0.02	18.00	9.97 ± 0.02	10.01	4.94 ± 0.01	4.99	2.95 ± 0.03	2.97
**FWHM (cm)**	0.47 ± 0.02	NA	0.45 ± 0.01	NA	0.38 ± 0.02	NA	0.27 ± 0.03	NA
**W_80_ (mm)**	3.23 ± 0.02	3.12	3.24 ± 0.01	2.58	2.41 ± 0.03	2.39	1.17 ± 0.05	2.34
**Shift@R_50_ (mm)**	0.1 ± 0.03	NA	0.36 ± 0.01	NA	0.20 ± 0.01	NA	0.47 ± 0.04	NA

## Data Availability

The original contributions presented in the study are included in the article/Supplementary Material, further inquiries can be directed to the corresponding author.

## Supplementary Materials

The following supporting information can be downloaded at: https://www.mdpi.com/article/10.3390/nano14201643/s1. Figure S1: Chemical structure representation of PVA/PFB NDs. Figure S2: (a) Schematic representation of the in-vitro irradiation setup of size-sorted PVA/PFB NDs phantoms to C-ions radiation @ 37 °C (Sci. Rep. 12, 8012 (2022). https://doi.org/10.1038/s41598-022-11524-x). (b) Schematic illustration of the depth-resolved imaging scan of phantoms along the lateral width (Y axis) of the PMMA container (green arrow), and of the acoustic window considered for each image (the blue double arrow line indicates extent of the lateral length of the acoustic window, i.e., 3 cm). The center of the probe was aligned with the middle of the internal length of the phantom container (red triangle), i.e., parallel to the beam direction. Figure S3: Example of nanodroplets (NDs-S, i.e., NDs5000) counting using pixel maxima detection with Fiji-ImageJ freeware (left image) and Laser-scanning confocal microscopy image of NDs labeled with RBITC fluorescent dye (right image). The labeling with the dye is achieved by incubating NDs with 2 μL of RBITC (1 mg/mL in DMSO) for 1 h at 5 °C, and then extensively washed by multi-step centrifugation to remove the excess). Figure S4: DSC heating thermograms of the size sorted PVA/PFB NDs. The scan was performed using TA Q2000 differential scanning calorimeter (TA Instruments, Vimodrone, MI, Italy): 30 μL of NDs pellet were filled into a high volume-pressure aluminum pan sealed with a lid. The thermograms were recorded under a nitrogen flow of 50 mL/min and within a temperature range of 5–110 °C at a heating rate of 3 °C/min. Figure S5: (a) Example pre-irradiation US imaging (MI = 0.1, fc = 7.5 MHz) of NDs phantoms containing 400 nm NDs (left) and 900 nm NDs (right). These images were recorded after incubation at 37 °C and prior to exposure to C-ions (180 mm, 1 Gy); the NDs concentration in both samples is 8 × 10^5^ NDs/ml. (b) Overlay of the L-NDs (25 min sonication) vaporization profiles post-exposure at doses from 0.5 to 4 Gy and the Bragg curve (measured with “peakfinder”) corresponding to 180 mm range (312 MeV/u). Figure S6: Evaluation of the concentration effect on the NDs response at low doses of C-ions (180 mm, 312 MeV/u): (a) US images (MI 0.1, fc = 7.5 MHz) of L-NDs phantoms post 0.1-Gy irradiation with NDs concentrations of ≈8.4 × 10^5^, 2 × 10^6^ and 4 × 10^6^ NDs/mL. (b) Histogram of the echocontrast profile peak integrals relative to NDs vaporization at the BP at the different tested concentrations post 0.1 Gy. The inset figure shows the linear correlation between NDs response at 0.1 Gy and their concentration. (c) Comparison of echocontrast peak integrals when varying NDs concentration and C-ions dose with the same factor (i.e., for 0.1 Gy of C-ion exposure, the NDs concentration is 5 times higher than the concentration tested for 0.5 Gy at the same range). (d) Pre-and post-0.1 Gy US images of S-NDs phantom (25 min sonication; C = 2 × 10^6^ NDs/mL) and the corresponding grey-value vaporization (the pre-irradiation background signal is subtracted). Figure S7: Quantification of the vaporization signals of S-NDs (obtained after 15 min sonication; C ≈ 10^6^ NDs/ml) exposed to 1 Gy C-ions as a function of the initial C-ions energy (a) and corresponding values of particles count/spot (b). Figure S8: (a,b) Evaluation of the peaks integrals from the average grey-value vaporization profiles of L-NDs and S-NDs, respectively, as a function of C-ions dose at 50 mm range. (c) Overlay of normalized echocontrast vaporization profiles at 0.1 Gy of L-NDs and S-NDs (25 min sonication, C = 2–4 × 10^6^ NDs/mL) and Bragg curve (measured with PeakFinder) at 180 mm. (d) Normalized vaporization profiles of L-NDs (25 min sonication; C = 2 × 10^6^ NDs/mL) at doses form 0.5 to 4 Gy and the Bragg curve (measured with “peakfinder”) corresponding to 50 mm range. (e) Overlay example of the S-NDs (15 min sonication, C = 10^6^ NDs/mL)) vaporization profile (post-1Gy exposure) and the Bragg curve at 180 mm range. (f) Echocontrast profile of S-NDs (C = 2 × 10^6^ NDs/mL) post-1Gy SOBP irradiation from 160 mm to 180 mm (inset image is the post-irradiation full-length US scan @ MI = 0.1 and fc = 7.5 MHz). The reported NDs concentrations refer to the phantom content. Figure S9: (a) Gaussian-fit of the multiple-paint vaporization profile of L-NDs (25 min, C ≈ 10^6^ NDs/mL), extracted from US imaging scan, after exposure to 2 Gy of C-ions with selected depths between 126–174 mm (the green lines correspond to the single Gaussians for each peak). (b,c) the measured average areas and FWHM of the vaporization peaks from the US. The red circle indicates the presence of artifact from the image combinations to cover the full phantom length. Figure S10: Post-irradiation (5 Gy, 400 MeV/u single spot) US images (MI = 0.7, fc = 7.5 MHz) of S-NDs phantom acquired with the US trasnducer positioned in parallel and perpendicular directions respect to the C-ions beam and the corresponding echocontrast profiles obtained with the two projections. Table S1: Summary of C-ions irradiation settings. Table S2: Mean diameter Summary of size-sorted PVA/PFB NDs samples. Table S3: Analyses of the S-and L-NDs (obtained with 25 min of soication; C ≈ 10^6^ NDs/mL) vaporization profiles post-1Gy at 100 mm and 180 mm ranges in comparison to the measured Bragg peaks (via PeakFinder). The results derive from a Gaussian fit of the vaporized NDs peaks.
