# Supplementary material for: Size-Sorted Superheated Nanodroplets for Dosimetry and Range Verification of Carbon-Ion Radiotherapy
Source: Nanomaterials (Basel). 2024 Oct 13;14(20):1643. doi: 10.3390/nano14201643 (PMC11509999; doi:10.3390/nano14201643)
Supplement: Supplementary file 1 [file nanomaterials-14-01643-s001.zip › nanomaterials-3220180-supplementary.pdf]

Supporting Information

Size-Sorted Superheated Nanodroplets for Dosimetry and Range Verification of Carbon-Ion Radiotherapy

Yosra Toumia [1,2]*, Marco Pullia [3], Fabio Domenici [1,2], Alessio Mereghetti [3], Simone Savazzi [3], Michele Ferrarini [3], Angelica Facoetti [3], Gaio Paradossi [1,2]

[1]*National Institute for Nuclear Physics, INFN sez. Roma Tor Vergata, 00133 Rome, Italy*
[2]*Department of Chemical Science and Technologies, University of Rome Tor Vergata, 00133 Rome, Italy*
[3]*Fondazione CNAO, National Center for Oncological Hadrontherapy, 27100, Pavia, Italy*

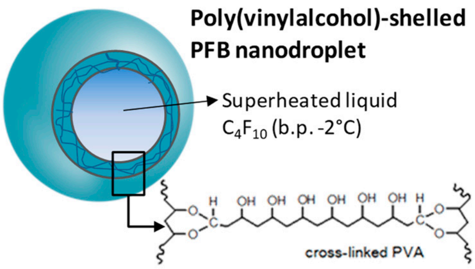

Figure S1. Chemical structure representation of PVA/PFB NDs

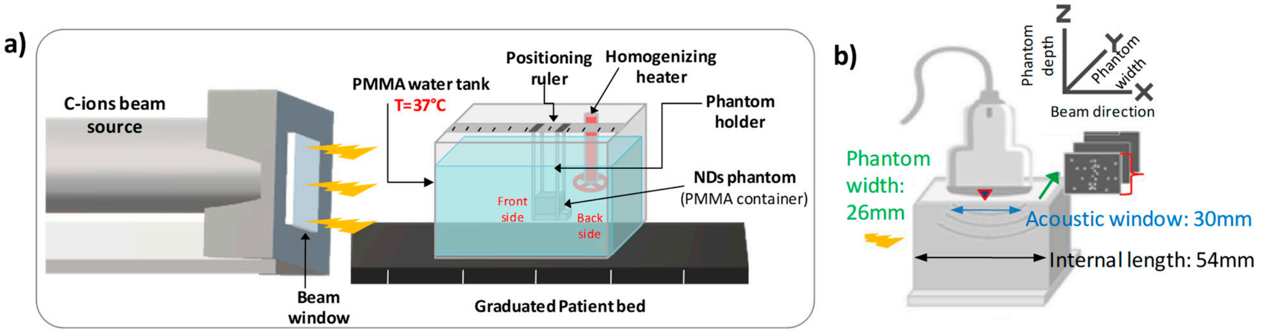

Figure S2. a) Schematic representation of the in-vitro irradiation setup of size-sorted PVA/PFB NDs phantoms to C-ions radiation @ 37°C (*Sci. Rep.* 12, 8012 (2022). https://doi.org/10.1038/s41598-022-11524-x). b) Schematic illustration of the depth-resolved imaging scan of phantoms along the lateral width (Y axis) of the PMMA container (green arrow), and of the acoustic window considered for each image (the blue double arrow line indicates extent of the lateral length of the acoustic window, i.e. 3 cm). The center of the probe was aligned with the middle of the internal length of the phantom container (red triangle), i.e. parallel to the beam direction.

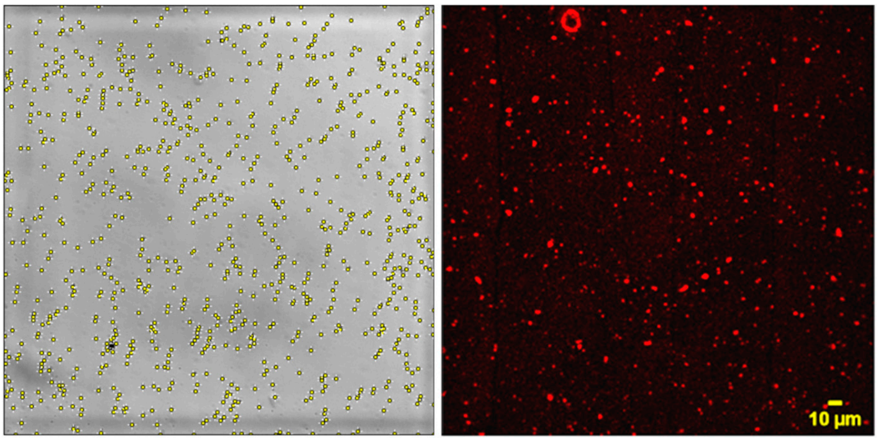

Figure S3 Example of nanodroplets (NDs-S, i.e. NDs5000) counting using pixel maxima detection with Fiji-ImageJ freeware (left image) and Laser-scanning confocal microscopy image of NDs labeled with RBITC fluorescent dye (right image). The labeling with the dye is achieved by incubating NDs with 2µl of RBITC (1mg/ml in DMSO) for 1hour at 5°C, and then extensively washed by multi-step centrifugation to remove the excess).

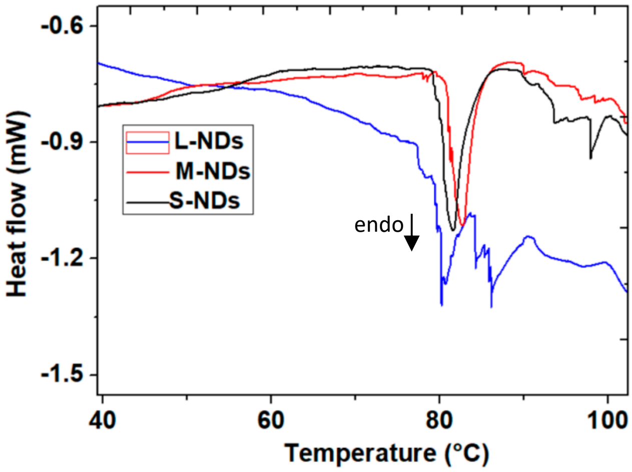

Figure S4 DSC heating thermograms of the size sorted PVA/PFB NDs. The scan was performed using TA Q2000 differential scanning calorimeter (TA Instruments, MI, Italy): 30 µl of NDs pellet were filled into a high volume-pressure aluminum pan sealed with a lid. The thermograms were recorded under a nitrogen flow of 50 ml/min and within a temperature range of 5-110 °C at a heating rate of 3 °C/min.

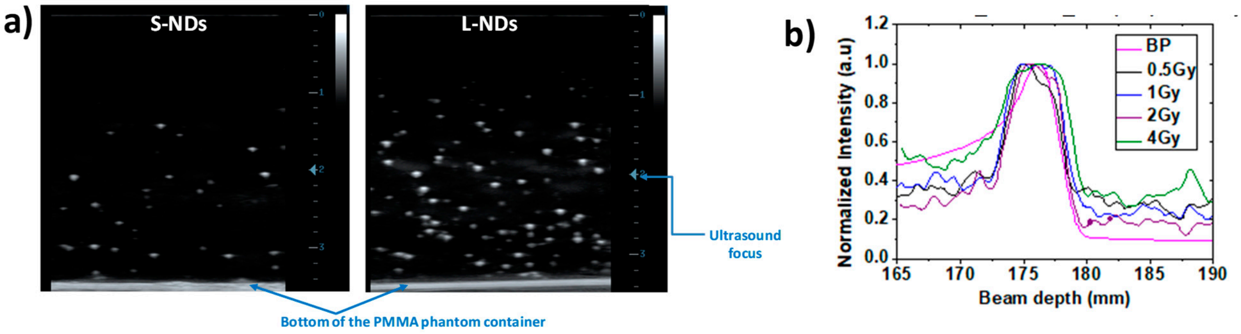

Figure S5. a) Example pre-irradiation US imaging (MI=0.1, f_c= 7.5 MHz) of NDs phantoms containing 400 nm NDs (left) and 900 nm NDs (right). These images were recorded after incubation at 37°C and prior to exposure to C-ions (180mm, 1Gy); the NDs concentration in both samples is $8{\times}10^5$ NDs/ml. b) Overlay of the L-NDs (25 min sonication) vaporization profiles post-exposure at doses from 0.5 to 4Gy and the Bragg curve (measured with "peakfinder") corresponding to 180 mm range (312MeV/u).

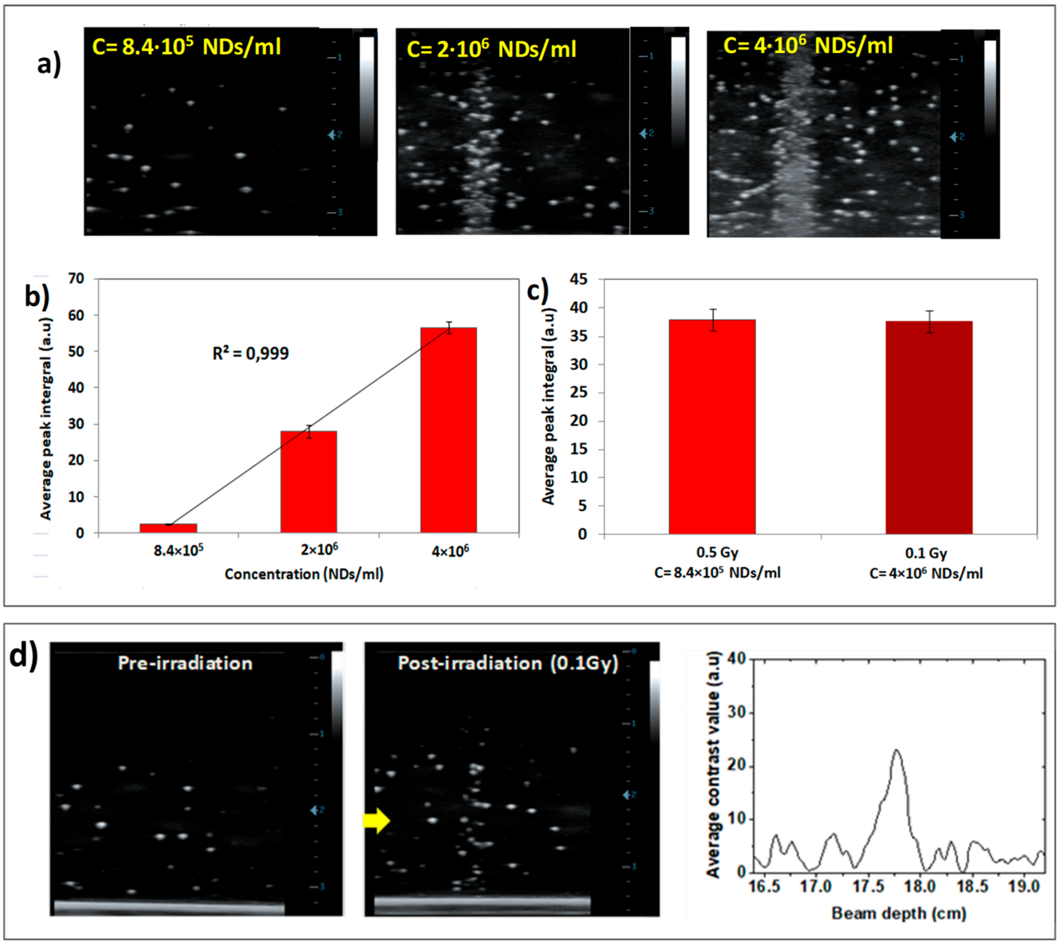

Figure S6. Evaluation of the concentration effect on the NDs response at low doses of C-ions (180mm, 312 MeV/u): a) US images (MI 0.1, f_c=7.5 MHz) of L-NDs phantoms post 0.1-Gy irradiation with NDs concentrations of ≈8.4×10^5, 2×10^6 and 4×10^6 NDs/ml. b) Histogram of the echocontrast profile peak integrals relative to NDs vaporization at the BP at the different tested concentrations post 0.1 Gy. The inset figure shows the linear correlation between NDs response at 0.1 Gy and their concentration. c) Comparison of echocontrast peak integrals when varying NDs concentration and C-ions dose with the same factor (i.e. for 0.1 Gy of C-ion exposure, the NDs concentration is 5 times higher than the concentration tested for 0.5 Gy at the same range). d) Pre-and post-0.1Gy US images of S-NDs phantom (25 min sonication; C=2×10^6 NDs/ml) and the corresponding grey-value vaporization (the pre-irradiation background signal is subtracted).

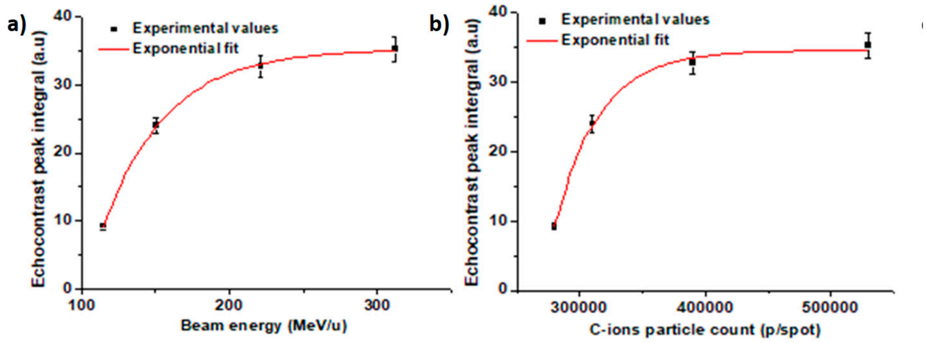

Figure S7. Quantification of the vaporization signals of S-NDs (obtained after 15 min sonication; C≈ 10^6 NDs/ml) exposed to 1Gy C-ions as a function of the initial C-ions energy (a) and corresponding values of particles count/spot (b).

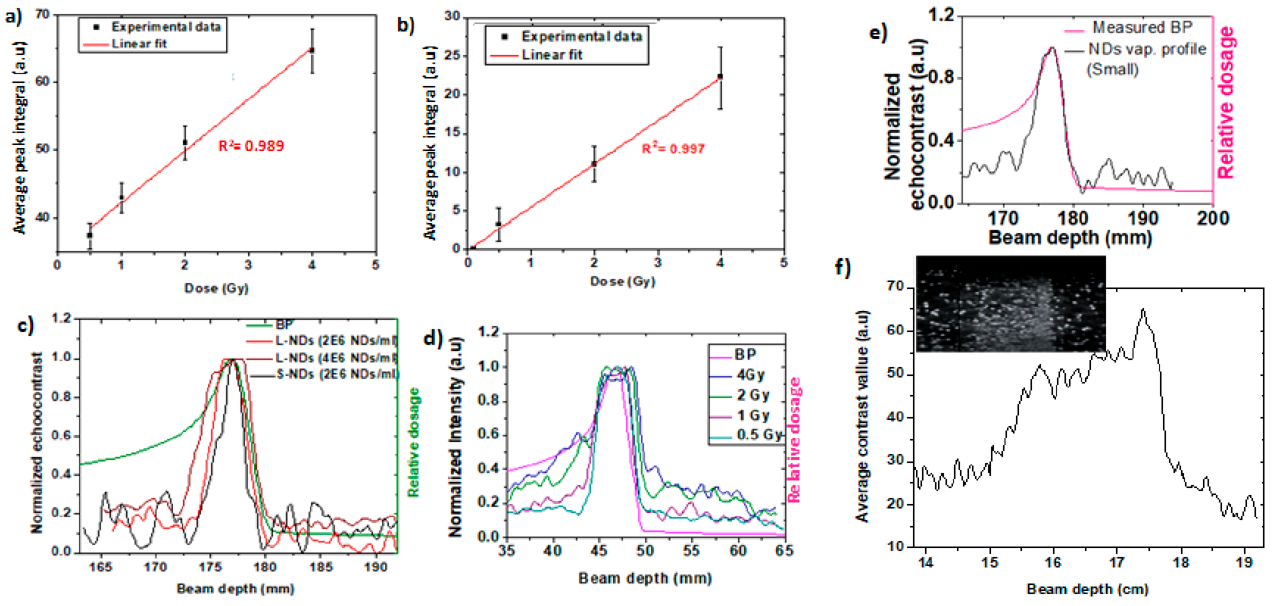

Figure S8. a) and b) Evaluation of the peaks integrals from the average grey-value vaporization profiles of L-NDs and S-NDs, respectively, as a function of C-ions dose at 50mm range. c) Overlay of normalized echocontrast vaporization profiles at 0.1 Gy of L-NDs and S-NDs (25 min sonication, C= 2-4×10^6 NDs/ml) and Bragg curve (measured with PeakFinder) at 180 mm. d) Normalized vaporization profiles of L-NDs (25 min sonication; C=2×10^6 NDs/ml) at doses form 0.5 to 4Gy and the Bragg curve (measured with "peakfinder") corresponding to 50 mm range. e) Overlay example of the S-NDs (15min sonication, C= 10^6 NDs/ml)) vaporization profile (post-1Gy exposure) and the Bragg curve at 180 mm range. f) Echocontrast profile of S-NDs (C= 2 ×10^6 NDs/ml) post-1 Gy SOBP irradiation from 160 mm to 180 mm (inset image is the post-irradiation full-length US scan @ MI=0.1 and f_c= 7.5 MHz). The reported NDs concentrations refer to the phantom content.

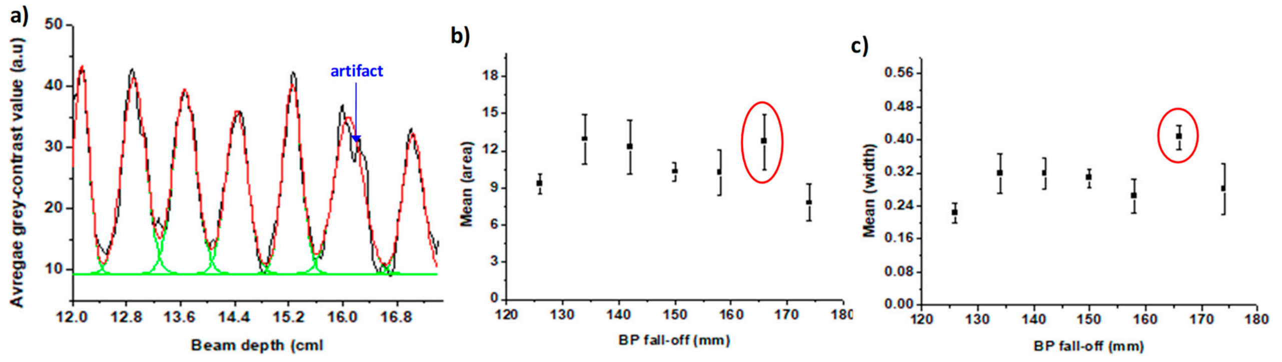

Figure S9 a) Gaussian-fit of the multiple-paint vaporization profile of L-NDs (25min, C≈.10^6 NDs/ml.), extracted from US imaging scan, after exposure to 2 Gy of C-ions with selected depths between 126-174 mm (the green lines correspond to the single Gaussians for each peak). b) and c) the measured average areas and FWHM of the vaporization peaks from the US. The red circle indicates the presence of artifact from the image combinations to cover the full phantom length.

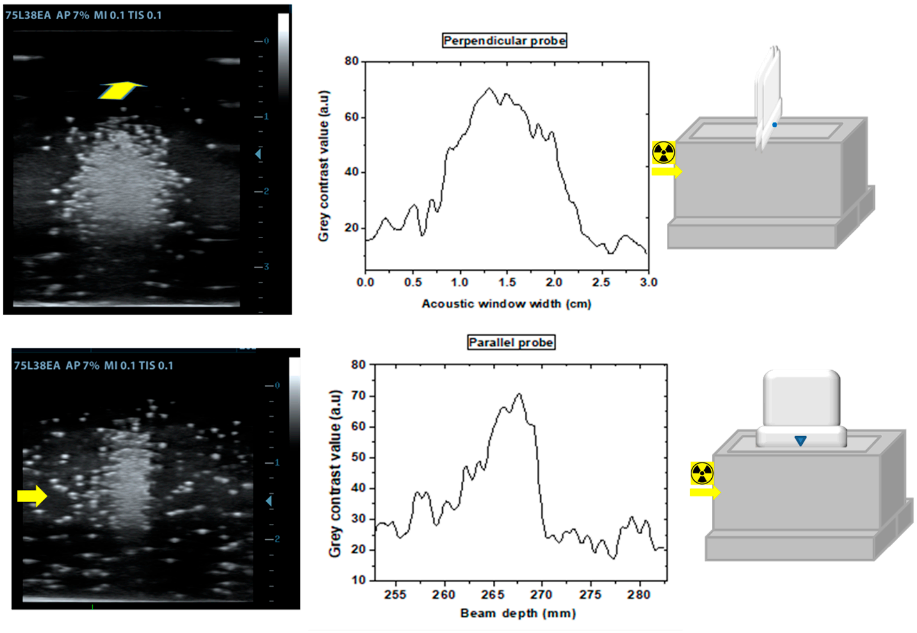

Figure S10. Post-irradiation (5Gy, 400 MeV/u single spot) US images (MI=0.7, f_c=7.5 MHz) of S-NDs phantom acquired with the US trasnducer positioned in parallel and perpendicular directions respect to the C-ions beam and the corresponding echocontrast profiles obtained with the two projections.

Table S1. Summary of C-ions irradiation settings

Irradiation plan	Nominal energy (MeV/u)	Range (mm)	Phantom position (cm)	Dose @BP (Gy)	C-ions count/spot	Temperature (°C)
Single BP	311.85	180	15	0.1	5.3E4	37
Single BP	311.85	180	15	0.5	2.65E5	37
Single BP	311.85	180	15	1	5.3E5	37
Single BP	311.85	180	15	2	1.06E6	37
Single BP	311.85	180	15	4	2.09E6	37
Single BP	221.45	100	7	1	3.9E5	37
Single BP	150.71	50	2	0.1	3E4	37
Single BP	150.71	50	2	0.5	1.5E5	37
Single BP	150.71	50	2	1	3.1E5	37
Single BP	150.71	50	2	2	6.2E5	37
Single BP	150.71	50	2	4	1.23E6	37
Single BP	115.23	30	0.4	1	2.8E5	37
SOBP	290.82-311.85	160-180	14	1	7.5E7-1E9	37
Multi-energy (n=7; d=8mm)	252.84-305.63	126-174	11.8	2	1.06E6	37
Spot	399	270	24	5	8.2E6	37

Table S2 Mean diameter Summary of size-sorted PVA/PFB NDs samples

Sample production parameters	Mean diameter (nm)		Polydispersity index (PDI)
PVA 2% (w/v), 15 min sonication	L-NDs (1000rpm)	850±85	0.09
	M-NDs (2500rpm)	700±52	0.14
	S-NDs (5000rpm)	450±38	0.04
PVA 2% (w/v), 25 min sonication	L-NDs (1000rpm)	810±170	0.9*
	M-NDs (2500rpm)	656±28	0.8*
	S-NDs (5000rpm)	400±35	0.27

(*)High PDI are believed to be due to excessive formation of bubbles in the cuvette walls.

Table S3. Analyses of the S-and L-NDs (obtained with 25 min of soication; C≈10^6NDs/ml) vaporization profiles post-1 Gy at 100mm and 180mm ranges in comparison to the measured Bragg peaks (via PeakFinder). The results derive from a Gaussian fit of the vaporized NDs peaks.

	100 mm			180mm		
	**BP**	**L-NDs**	**S-NDs**	**BP**	**L-NDs**	**S-NDs**
**X_c (mm)**	96.90	96.93	96.79	176.93	176.65	177.16
**X_{end}(mm)**	100	100.32	100.40	180	179.74	180.54
**Peak area**	NA	197.5	84.29	NA	222.4	178.4
**FWHM (mm)**	NA	3.34±0.07	2.87±0.07	NA	3.53±0.09	3.77±0.07
**W_{80}**	2.57	2.11±0.06	1.34±0.04	3	2±0.06	2.4±0.05
**Shift @R_{50} (mm)**	NA	0.11	0.28	NA	0.10	0.2
